# Arch- and Morphology-Related Variation in Early Marginal Bone Remodeling of Monolithic Zirconia Full-Arch Prostheses: An Exploratory Prospective Cohort Study

**DOI:** 10.3390/bioengineering13080933

**Published:** 2026-08-18

**Authors:** Luis Carlos Garza, Eduardo Crooke, Marta Vallés, Joan Soliva, Xavier Rodríguez, Miguel Roig

**Affiliations:** 1 Department of Restorative Dentistry, Universitat Internacional de Catalunya, 08195 Barcelona, Spain; mvalles@uic.es (M.V.); jsoliva@uic.es (J.S.); mroig@uic.es (M.R.); 2 Private Practice, 29016 Málaga, Spain; 3 Department of Oral Surgery, Universitat Internacional de Catalunya, 08195 Barcelona, Spain; xrodriguez@uic.es

**Keywords:** monolithic zirconia, full-arch implant-supported prosthesis, marginal bone remodeling, mandibular morphology, digital dentistry

## Abstract

Whether arch-specific anatomical configuration affects early peri-implant bone behavior in monolithic zirconia full-arch prostheses is unclear. This prospective cohort study evaluated 40 edentulous patients with 49 monolithic zirconia full-arch prostheses (25 maxillary, 24 mandibular; 308 implants). MBR and patient-reported outcomes were recorded at baseline and 12 months; mandibular morphology (U- vs. V-shaped) was classified on CBCT, and inter-implant distance and cantilever length were measured on CAD designs. Because 9 of 40 patients contributed both arches, the primary MBR comparison used a linear mixed-effects model; morphology and geometry associations were exploratory (ANOVA; Pearson correlation). Implant and prosthetic survival were 99.4% and 97.9%. Mean MBR was higher in the mandibular (0.86 ± 0.06 mm) than the maxillary arches (0.73 ± 0.02 mm); the mixed-effects model estimated this difference as β = 0.13 mm (95% CI: 0.10–0.16; *p* < 0.001). V-shaped mandibles (0.91 ± 0.04 mm) showed higher MBR than U-shaped mandibles (0.81 ± 0.04 mm; mean difference 0.10 mm; *p* < 0.001), and inter-implant distance correlated negatively with MBR in the mandible (r = −0.70, *p* < 0.001) but not in the maxilla (r = 0.01, *p* = 0.97). Patient-reported outcomes improved markedly in both arches; two minor chipping events occurred, both in V-shaped mandibles. Monolithic zirconia full-arch prostheses showed favorable short-term outcomes in both arches. Arch- and mandibular-morphology-related differences in early MBR were statistically robust and highly consistent in standardized effect size, although the absolute differences were numerically modest; these findings suggest an association between anatomical configuration and early bone remodeling under rigid, full-arch monolithic zirconia frameworks that warrants further confirmation.

## 1. Introduction

Digital workflows and high-strength ceramic materials have expanded the indications for full-arch implant rehabilitation. Among available restorative options, monolithic zirconia prostheses have gained widespread clinical acceptance due to their favorable mechanical strength, dimensional stability, biocompatibility, and reduced incidence of veneering-related complications [1,2]. Clinical studies and retrospective cohorts have consistently reported high implant and prosthetic survival rates, predictable short-term performance, and marked improvements in patient-reported outcomes, consolidating monolithic zirconia as a reliable solution for complete-arch rehabilitation [3].

From a biological perspective, early marginal bone remodeling around implants supporting zirconia full-arch prostheses has generally been reported within ranges considered physiologically acceptable during the first year of function. However, most available studies have evaluated marginal bone remodeling (MBR) as a global outcome, without stratifying results by arch or exploring potential anatomical or geometric modulators. As a result, current evidence largely reflects average behavior rather than context-specific bone adaptation.

In parallel, the literature has tended to assume that standardized surgical protocols, digital planning, and rigid prosthetic frameworks minimize anatomical variability between the maxilla and mandible. This assumption has contributed to the perception that bone remodeling patterns are broadly comparable across arches when similar restorative concepts are applied. This study tests this assumption rather than accepting it.

The mandible differs from the maxilla in cortical distribution, trabecular density, vascularization, and functional biomechanics, and exhibits elastic deformation during functional loading (mandibular flexure), influenced by muscular activity, bone morphology, and arch form [4,5,6]. In contrast, the maxilla demonstrates negligible functional deformation and a different pattern of load dissipation [7]. When a rigid, continuous, multi-implant-supported framework such as monolithic zirconia is connected across the mandibular arch, these biomechanical differences may have implications for load transfer at the implant–bone interface: zirconia’s high elastic modulus (≈200 GPa) limits its capacity to absorb or dissipate strain, so that flexural movement of the mandible during function is transmitted more directly as stress to the implant–bone interface than would occur with a more resilient framework material. Whether this translates into measurable differences in early bone remodeling, however, has not been established and is the empirical question addressed here. Beyond this rigid-framework paradigm, recent advances in additive manufacturing have also enabled biomimetic scaffold architectures and functionalized composite biomaterials that actively modulate cellular response and bone remodeling at the implant–bone interface, representing a complementary line of bioengineering research to the conventional digital workflows and zirconia materials discussed above [8,9].

Despite extensive documentation of digital accuracy [7,10,11,12], prosthetic precision, and patient satisfaction [13,14,15] associated with zirconia full-arch rehabilitations, direct clinical comparisons of early marginal bone remodeling between maxillary and mandibular zirconia full-arch prostheses remain scarce, and evidence addressing whether mandibular anatomical configuration influences early bone behavior under otherwise identical surgical and prosthetic conditions is even more limited [16,17].

Most previous clinical studies have not incorporated systematic assessments of arch morphology or implant distribution geometry into their analytical frameworks. Variables such as mandibular arch form, inter-implant distance, or spatial implant configuration have rarely been examined as potential contributors to early bone remodeling. This methodological gap restricts the ability to discern whether observed variations in marginal bone remodeling reflect biological variability alone, an interaction between anatomy, geometry, and prosthetic rigidity, or chance.

Accordingly, this investigation does not aim to incrementally confirm the general effectiveness of monolithic zirconia full-arch prostheses, which is already well established. Instead, it evaluates mandibular morphology and arch geometry as clinically accessible anatomical factors potentially associated with early marginal bone remodeling, treating the maxilla as a low-deformation reference and the mandible as a structure with potentially higher biomechanical variability.

Therefore, the objectives of this prospective clinical study were to compare 12-month MBR and clinical outcomes of monolithic zirconia full-arch implant-supported prostheses placed in the maxilla and mandible as the primary aim, and to explore, as a secondary and hypothesis-generating aim, whether mandibular morphology and implant distribution geometry are associated with early MBR. The null hypothesis was that no differences in early MBR would be observed between arches and between different mandibular morphologies.

## 2. Materials and Methods

### 2.1. Study Design and Ethical Approval

This study was designed as a prospective observational cohort study conducted under routine clinical conditions. The study did not involve randomized control groups or protocol-driven therapeutic interventions beyond the standard of care. Ethical approval was obtained from the Institutional Review Board of Universitat Internacional de Catalunya (REST-ECL-2020-02). This study is reported in accordance with the STROBE guidelines for observational studies (Supplementary File S1).

For transparency purposes, the study protocol was registered retrospectively on ClinicalTrials.gov (NCT07323290). Due to the timing of registration, the study should be interpreted as an observational clinical investigation rather than a clinical trial. All participants provided written informed consent prior to inclusion.

The study was designed to evaluate early marginal bone remodeling under standardized surgical and prosthetic conditions, while allowing exploratory assessment of arch-specific anatomical and geometric factors. The maxilla was considered a low-deformation reference structure, whereas the mandible was analyzed as a biomechanically heterogeneous arch with potential morphology-related variability.

### 2.2. Study Population

Patients were consecutively recruited within the defined study period from a university-based clinical setting. Inclusion criteria were age ≥ 50 years, adequate bone volume for implant placement, and non-smokers or light smokers (<10 cigarettes/day). Exclusion criteria comprised diagnosis of bruxism or other severe parafunctional habits, bisphosphonate therapy, uncontrolled systemic disease, history of head and neck radiotherapy, or any contraindication to implant surgery or immediate loading protocols.

During the broader period in which this treatment protocol was offered clinically, 56 patients received monolithic zirconia full-arch implant-supported prostheses (72 prostheses, 432 implants) meeting the general treatment indications described above. Of these, 40 patients (49 prostheses, 308 implants) were treated within the defined study period and underwent the standardized prospective 12-month assessment protocol described in this manuscript, constituting the present study cohort; all 40 completed the full 12-month follow-up, and none were lost to follow-up. The remaining 16 patients were treated outside the defined study period and were not assessed under the standardized prospective protocol; they were therefore not eligible for inclusion in the present analysis. Granular, per-patient screening and exclusion data (e.g., number declining participation, individual reasons for exclusion within the defined study period) were not prospectively maintained in structured form and cannot be reported.

Baseline implant- and prosthesis-related variables, including number of implants per arch, anterior/posterior implant distribution, implant length and diameter, insertion torque, immediate loading criteria fulfillment, presence of bone augmentation, opposing dentition characteristics, and relevant systemic/local risk factors, were prospectively recorded to characterize potential confounders between arches. Opposing dentition was recorded prospectively only at the broad categorical level of natural versus prosthetic (Table 1); more granular characteristics—including specific restorative material, occlusal scheme, and number of opposing functional units—were not captured as discrete study variables and could not be analyzed (Section 4.5, Twelfth).

### 2.3. Surgical and Prosthetic Procedures

All surgical procedures were performed by the same experienced surgeon following a fully guided, standardized digital workflow, minimizing inter-operator variability. Preoperative planning was based on cone-beam computed tomography (CBCT; i-CAT FLX, Imaging Sciences, Hatfield, PA, USA) and digital prosthetic planning software to determine optimal implant positioning, angulation, and distribution.

A mucoperiosteal flap was raised under local anesthesia, and implants were placed using a tooth- and bone-supported surgical guide. Nobel Parallel Ti-Ultra implants (Nobel Biocare AB, Göteborg, Sweden) were used for all patients and placed following the manufacturer’s recommended drilling protocol under copious irrigation. Multi-unit abutments (Nobel Biocare AB, Göteborg, Sweden) were subsequently inserted and torqued to 35 Ncm per manufacturer recommendations. Scan bodies (Avinent^®^ Implant System, Santpedor, Spain) were attached for postoperative intraoral scanning.

All implants were bone-level, tapered implants with a moderately rough, sandblasted, and acid-etched surface. Implant diameter and length were selected based on local bone availability and prosthetic planning.

Implants were inserted with a target insertion torque of ≥35 Ncm. Only implants achieving this threshold and demonstrating adequate primary stability were considered eligible for immediate loading.

Implants were placed at crestal or slightly subcrestal level according to bone quality and platform-switching principles, with care taken to maintain adequate inter-implant distance and prosthetic emergence profile. Implant positioning followed a standardized clinical and laboratory protocol described previously [18], aiming for a homogeneous placement position according to manufacturer recommendations and each site’s clinical situation; however, exact insertion depth was not recorded as a discrete study variable and could therefore not be analyzed statistically.

Immediate loading was performed when all of the following objective criteria were met: insertion torque ≥ 35 Ncm for all implants; absence of intraoperative complications; favorable implant distribution allowing a provisional restoration; and achievement of passive fit of the provisional prosthesis. When these criteria were fulfilled, a screw-retained PMMA provisional prosthesis was delivered within 48 h post-surgery. Implant Stability Quotient (ISQ) values via resonance frequency analysis were not obtained; primary stability was assessed exclusively through insertion torque, consistent with the surgical protocol adopted for this cohort. Provisional prosthesis design was individualized per patient within this standardized fully digital workflow, as previously described [19].

### 2.4. Prosthetic Protocol

**Figure 1:**
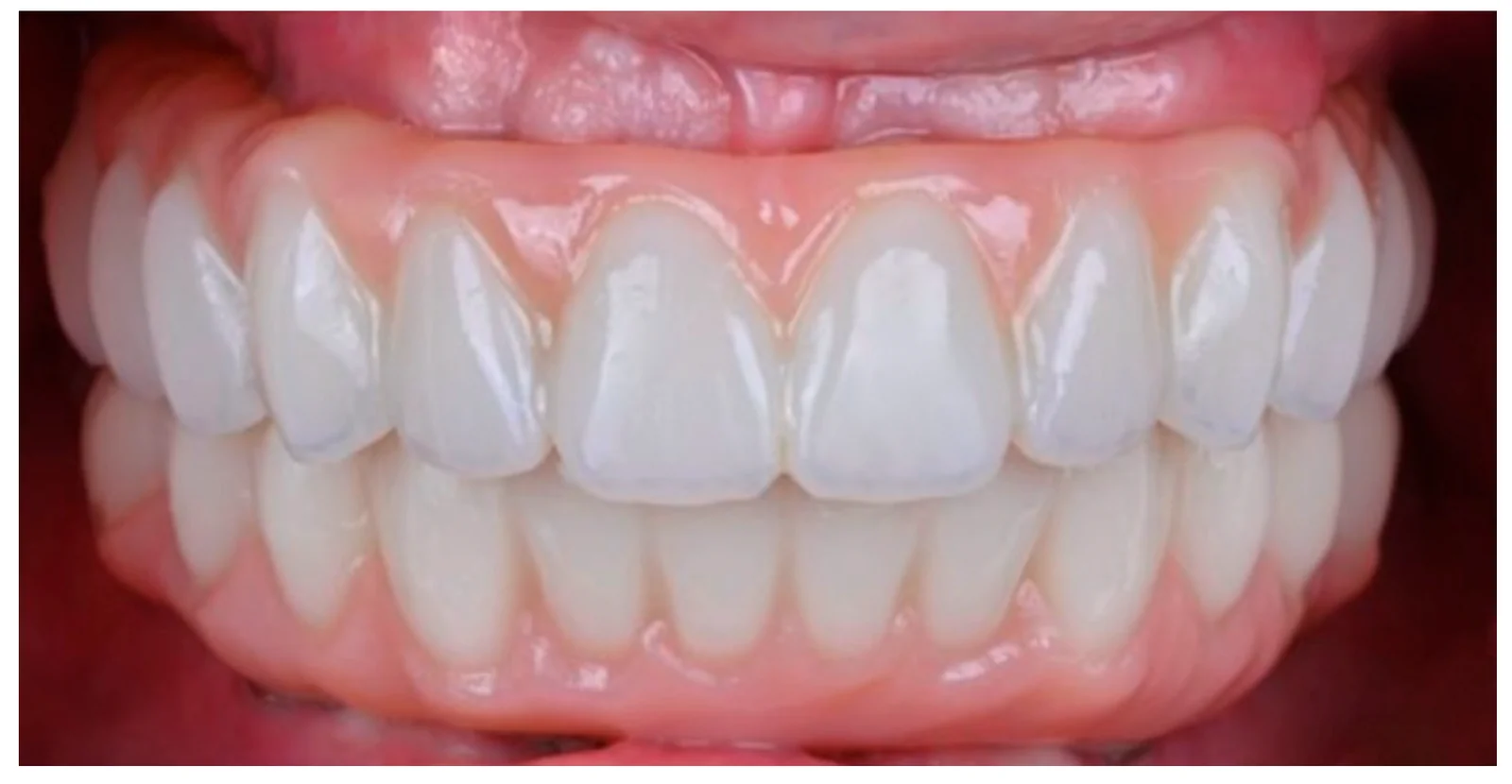
Monolithic zirconia full-arch prostheses in the maxilla and mandible.

After a 3-month healing period, definitive prostheses were fabricated using a fully digital CAD/CAM workflow, which ensured consistent implant-geometry measurement and minimized procedural variability. Definitive restorations consisted of screw-retained monolithic zirconia frameworks fabricated from Katana Zirconia pre-sintered blanks (Kuraray Noritake Dental Inc., Tokyo, Japan; 3Y-TZP), milled using a DWX-52D^®^ milling unit (DGSHAPE Corporation, Hamamatsu, Japan) and sintered according to manufacturer recommendations (Figure 1). No veneering porcelain was applied to functional areas.

The prosthetic design followed standardized parameters: (1) minimum framework thickness ≥ 3.0 mm in load-bearing areas; (2) connector cross-sectional area ≥ 12 mm^2^; and (3) rounded internal line angles to reduce stress concentration. Inter-implant distance and cantilever length were determined during the CAD phase and recorded for subsequent analysis. All definitive prostheses were screw-retained over multi-unit abutments; no cemented restorations were included.

Occlusion was adjusted to provide even bilateral contacts in maximum intercuspation, with light anterior guidance and avoidance of posterior interferences during lateral excursions.

### 2.5. Radiographic Assessment

Standardized periapical radiographs were obtained at baseline (prosthesis delivery) and at the 12-month follow-up using a CMOS digital sensor and a paralleling technique with customized positioning devices (Rinn XCP, Dentsply Sirona, Charlotte, NC, USA) to ensure reproducible angulation. Radiographs were taken using fixed exposure parameters, constant focus-to-sensor distance, and individualized positioning guides. Images were calibrated using the known implant thread pitch to correct for magnification.

Marginal bone levels were measured from the implant–abutment interface to the first bone-to-implant contact on the mesial and distal aspects using ImageJ version 1.54i software software (National Institutes of Health, Bethesda, MD, USA). The implant-level and arch-level aggregation procedure used to derive MBR is described in Section 2.7.

Two independent, calibrated examiners performed all measurements. Intra- and inter-examiner reliability were excellent (ICC = 0.94). Based on repeated measurements, the estimated measurement error was <0.1 mm; any discrepancy greater than 0.1 mm was reassessed jointly until consensus was reached.

### 2.6. Morphological and Geometric Assessment

This study was not designed to measure mandibular flexure or strain directly; morphology and geometry were instead evaluated as clinically accessible anatomical proxies potentially associated with early bone remodeling.

Mandibular morphology was classified on preoperative CBCT scans, acquired during initial digital treatment planning before implant placement, using a previously validated method [20,21]. Intercanine width was measured as the linear distance between the most prominent points of the right and left canine eminences, and intermolar width as the linear distance between the most prominent buccal points of the right and left first molar regions; both were measured on standardized axial CBCT slices at the level of the residual alveolar ridge crest. Classification was therefore based on alveolar ridge morphology rather than basal bone contour. The anterior arch angle was measured between lines connecting the central incisor region to each canine eminence. Mandibles with an intercanine/intermolar width ratio ≥ 0.80 and an anterior angle ≥ 120° were classified as U-shaped; mandibles with a ratio < 0.80 and an angle < 120° were classified as V-shaped.

Agreement between examiners was substantial (κ = 0.78). Given this level of agreement, a degree of non-differential misclassification cannot be excluded; such misclassification would be expected to attenuate, rather than inflate, the observed morphology-related differences. Borderline cases were resolved by consensus after joint review of the CBCT images by both examiners.

Inter-implant distance (linear distance between the two most distal implants in each arch) and cantilever length (distance from the most distal implant to the distal-most point of the prosthetic extension) were measured directly on the definitive CAD designs and analyzed independently to avoid collinearity.

### 2.7. Statistical Analysis

The implant was the unit of radiographic measurement. The arch, rather than the individual implant, was selected as the unit of analysis because the study’s central hypothesis concerns the behavior of a single rigid, continuous prosthetic framework spanning all implants within an arch. For each implant, mesial and distal marginal bone level measurements were averaged to obtain a single implant-level MBR value. Implant-level values were then averaged with equal weighting across all implants within each prosthetic arch to obtain the arch-level mean MBR used for the primary analysis; the number of implants per arch (mean ± SD, range) is reported in Table 1.

Of the 40 patients included, 9 (22.5%) contributed both a maxillary and a mandibular prosthesis, accounting for 18 of the 49 prostheses; arch-level observations were therefore not fully independent. The primary comparison of 12-month MBR between maxillary and mandibular arches was analyzed using a linear mixed-effects model with patient as a random intercept, fitted by restricted maximum likelihood, to account for this within-patient clustering. A sensitivity analysis was performed excluding the 9 dual-arch patients (retaining the 31 patients contributing a single arch) to confirm that the arch-level association was not driven solely by within-patient correlation.

A sample size calculation for this primary comparison indicated that a minimum of 18 arches per group was required to detect a 0.2 mm difference in MBR (SD 0.30 mm; α = 0.05; power = 80%); the final sample (25 maxillary, 24 mandibular arches) exceeded this threshold. This calculation was performed for the arch-level MBR comparison only. The associations between mandibular morphology, inter-implant distance, cantilever length, and MBR were exploratory, hypothesis-generating analyses; this study was not powered to establish causal or confirmatory relationships for these variables. Although the *p*-values obtained for the morphology and inter-implant-distance associations were well below the conventional 0.05 threshold, multiple subgroup and correlation analyses were performed, and formal confirmation in an independently powered, prospectively designed sample remains warranted; effect sizes and confidence intervals are emphasized alongside *p*-values for this reason.

Normality of continuous variables was confirmed using the Shapiro–Wilk test before parametric analyses. Morphology subgroup comparisons (U- vs. V-shaped mandibles) were analyzed using one-way ANOVA with Bonferroni correction. Pearson correlation coefficients were calculated to explore associations between geometric variables and MBR independently within each arch. Cohen’s d effect sizes were calculated for the primary arch-level comparison and for the mandibular morphology comparison from the reported means, standard deviations, and group sizes. Boxplots were used for graphical data visualization.

All statistical analyses were reviewed and validated by an independent statistician who was not involved in data collection. Descriptive statistics, the Shapiro–Wilk test, ANOVA, and Pearson correlations were performed using Statgraphics Centurion XIX (Statgraphics Technologies, The Plains, VA, USA); the linear mixed-effects model was fitted using R (version 4.1.1) with the lme4 package, and *p*-values were obtained using the lmerTest package with Satterthwaite’s approximation. A two-sided α = 0.05 was adopted for all inferential procedures.

## 3. Results

Forty edentulous patients were included and received a total of 49 monolithic zirconia full-arch implant-supported prostheses (Table 1). The cohort had a mean age of 62.3 ± 7.2 years (range, 50–75) and comprised 23 men (57.5%) and 17 women (42.5%). All analyses were performed at the arch level. Of these, 25 prostheses were placed in the maxilla and 24 in the mandible. All patients completed the 12-month follow-up, and no cases were lost to observation; data completeness for clinical, radiographic, and patient-reported outcomes was 100%.

A total of 308 implants were placed to support the full-arch prostheses. Two implants failed during the observation period, resulting in an implant survival rate of 99.4%. One prosthesis required replacement due to minor chipping, yielding a prosthetic survival rate of 97.9%. Survival outcomes were comparable between maxillary and mandibular arches (Table 2).

### 3.1. Marginal Bone Remodeling

Mean MBR increased over time in both arches. At the 12-month evaluation, mandibular arches showed a statistically significant higher mean MBR than maxillary arches (0.86 ± 0.06 mm vs. 0.73 ± 0.02 mm), an absolute difference of 0.13 mm (Cohen’s d ≈ 2.77, a large standardized effect size, although the absolute magnitude of the difference is modest). Accounting for within-patient clustering, a mixed-effects model confirmed a significant maxilla–mandible difference (β = 0.13 mm; 95% CI: 0.10–0.16; *p* < 0.001); the sensitivity analysis excluding the 9 dual-arch patients (31 single-arch patients, 31 arches) confirmed the findings (mean difference 0.11 mm; t = 7.01; *p* < 0.001), indicating a consistent and robust effect independent of within-patient clustering.

**Figure 2:**
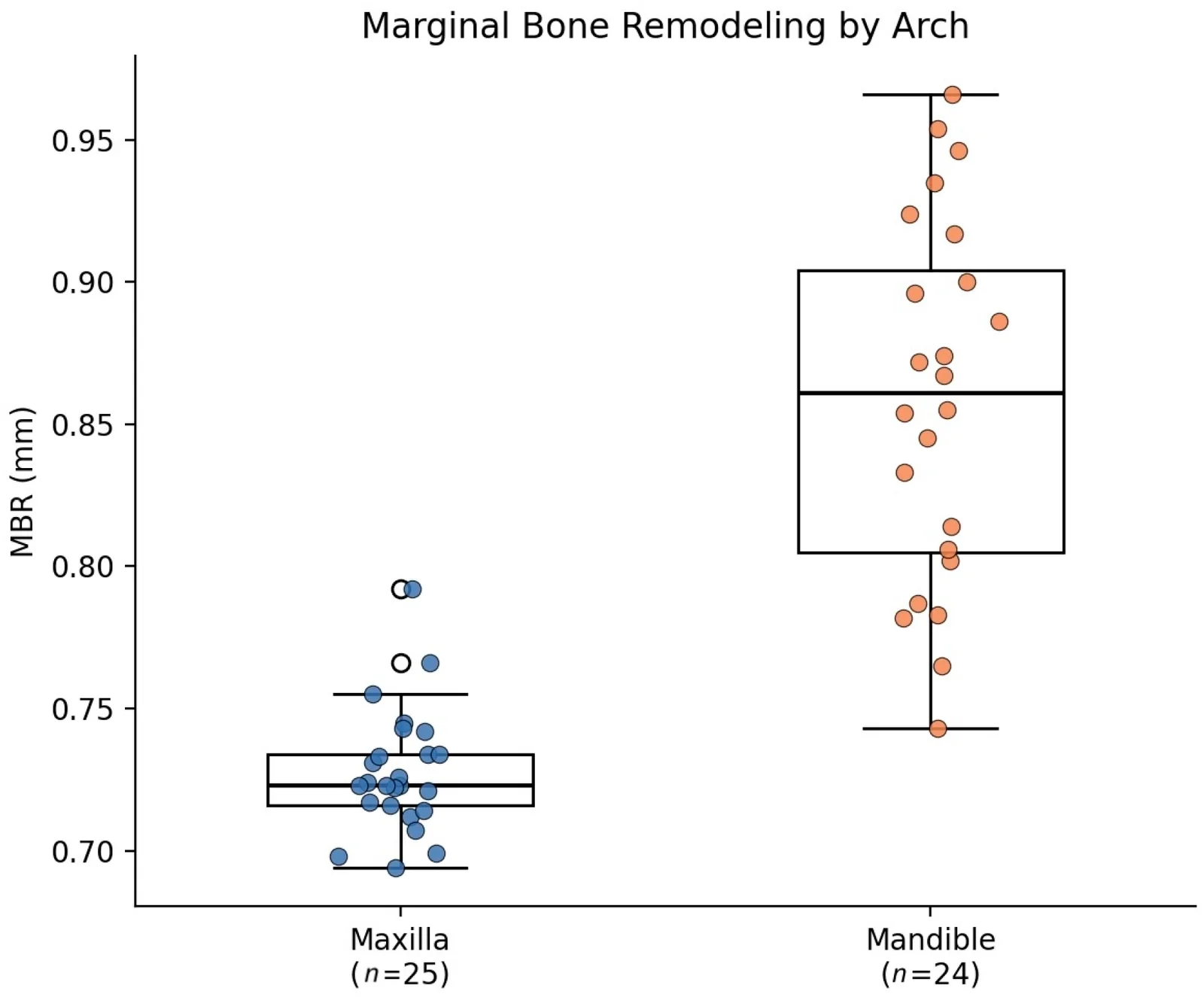
Marginal bone remodeling distribution comparing maxillary and mandibular arches. Boxplots indicate median, interquartile range, and 95% CI; individual data points are overlaid (jittered) to show the underlying distribution.

While the absolute difference of 0.13 mm remains below the 0.2 mm/year threshold conventionally used to define successful implant outcomes after the first year of function [22], its consistency across the primary and sensitivity analyses, together with its large standardized effect size, indicates a pattern unlikely to reflect chance alone, even though its absolute magnitude is modest and its clinical decisiveness at the individual-patient level remains to be established over longer follow-up and independent replication. Distribution of MBR values by arch is presented in Figure 2 and summarized in Table 3.

**Figure 3:**
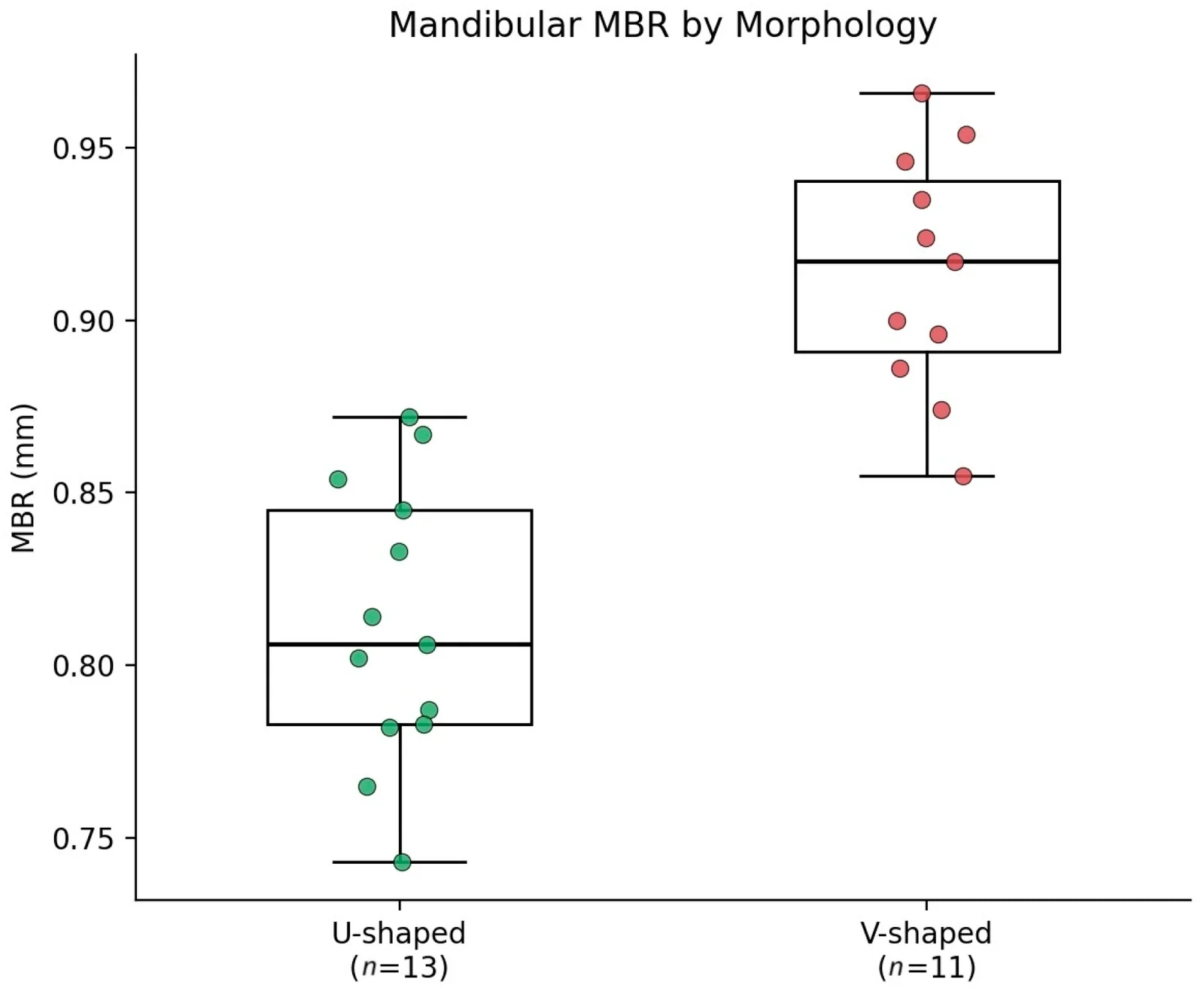
Mandibular 12-month marginal bone remodeling by morphology (U- vs. V-shaped). Boxplots show morphological differences; individual data points are overlaid (jittered) to show the underlying distribution.

Within the mandibular group, MBR differed according to morphological classification. U-shaped mandibles showed a mean MBR of 0.81 ± 0.04 mm (*n* = 13 arches), and V-shaped mandibles showed a mean MBR of 0.91 ± 0.04 mm (*n* = 11 arches). The one-way ANOVA estimated mean difference for this comparison was 0.10 mm (*p* < 0.001; Cohen’s d ≈ 2.69, a large standardized effect size), consistent with the difference obtained by simply subtracting the descriptive means in Table 4. Despite the modest subgroup sizes, the standardized effect size and statistical significance of this difference are consistent with a true association between mandibular morphology and early MBR, although the absolute difference (0.10 mm) is numerically small and confirmation in an independently powered, pre-specified sample remains warranted (Figure 3). Maxillary arches did not demonstrate morphology-dependent differences. Arch- and morphology-specific MBR values are reported in Table 4.

**Figure 4:**
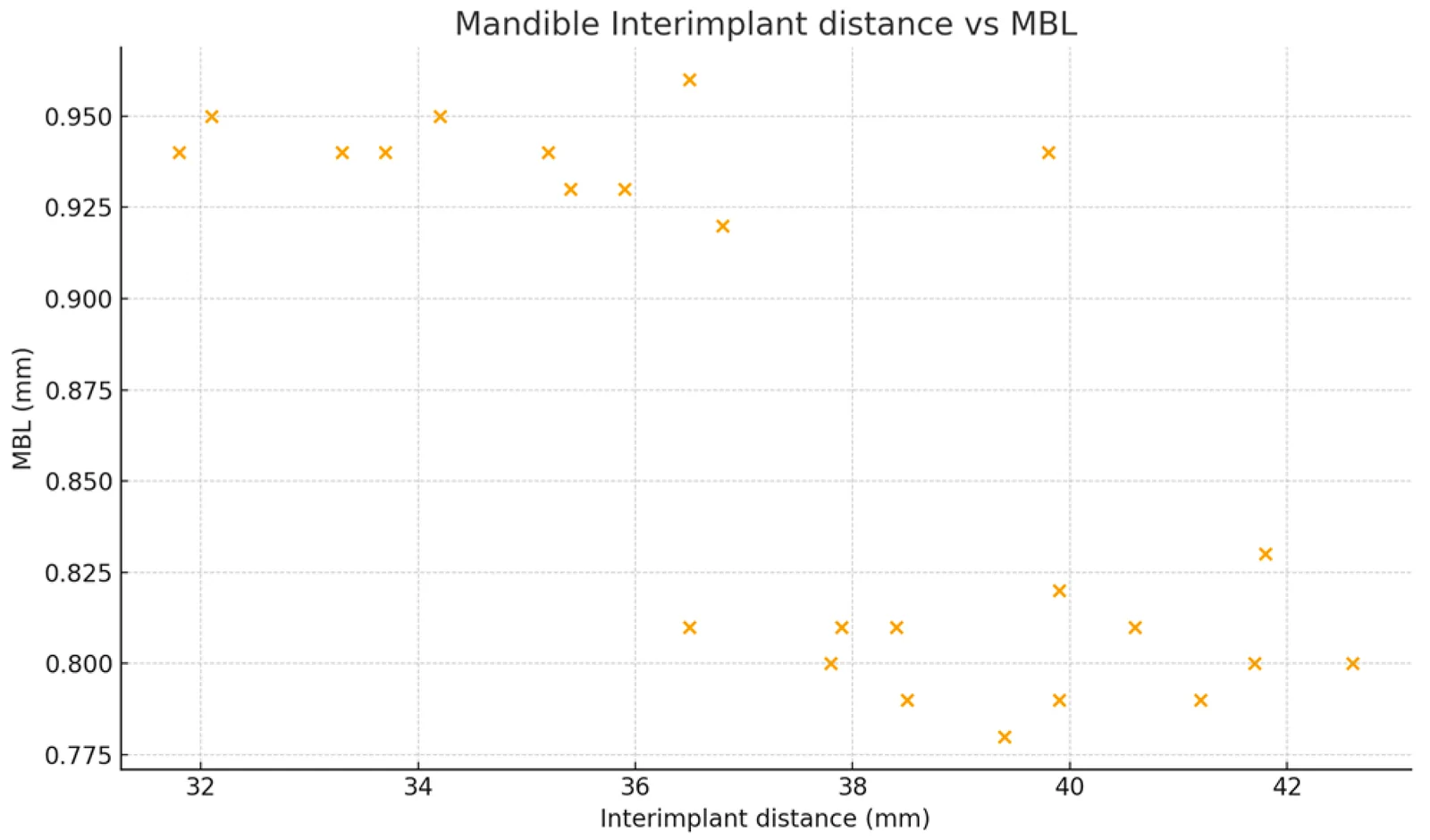
Correlation between inter-implant distance and 12-month marginal bone remodeling in the mandible. Linear regression line with 95% CI shown.

Mean inter-implant distance was greater in maxillary than in mandibular arches (41.2 ± 1.0 mm vs. 37.5 ± 3.1 mm). Within the mandible, U-shaped configurations showed larger inter-implant distances than V-shaped configurations (39.7 ± 1.8 mm vs. 35.0 ± 2.3 mm). Inter-implant distance correlated strongly and negatively with MBR in the mandible (r = −0.70, *p* < 0.001) but not in the maxilla (r = 0.01, *p* = 0.97); cantilever length did not correlate significantly with MBR in either arch. Detailed geometric measurements and correlations are presented in Figure 4 and summarized in Table 5.

### 3.2. Mechanical Complications

A total of two mechanical complications were recorded during follow-up, both consisting of minor chipping in mandibular V-shaped configurations. Cantilever lengths for these two prostheses were 11.4 mm and 12.7 mm, respectively, both above the mean for V-shaped mandibular configurations (10.5 ± 2.5 mm; Table 4), with the second case approaching one standard deviation above this mean. While the sample is too small (*n* = 2) to support statistical inference, this pattern is consistent with the hypothesis that longer cantilever extensions may contribute to localized mechanical overload in narrower, V-shaped mandibular arches. These events were anecdotal, and the study was not powered to assess complication risk.

No framework fractures or screw loosening events were observed. No mechanical complications were recorded in maxillary prostheses or in U-shaped mandibular arches. Mechanical complication rates by arch and morphology are summarized in Table 6.

### 3.3. Patient-Reported Outcomes

Patient-reported outcomes improved significantly from baseline to the 12-month follow-up. Mean OHIP-14 scores decreased from 52.3 ± 8.1 to 18.9 ± 4.3 (*p* < 0.001). Visual analogue scale (VAS) satisfaction increased from 4.1 ± 1.2 to 9.2 ± 0.7 (*p* < 0.001). Improvements were observed across both arches without statistically significant differences between maxillary and mandibular rehabilitations (Table 7).

## 4. Discussion

The present prospective clinical study evaluated early marginal bone remodeling around monolithic zirconia full-arch implant-supported prostheses and identified statistically consistent and highly significant differences, with large standardized effect sizes but numerically modest absolute magnitudes, between arches and within mandibular configurations. Both maxillary and mandibular rehabilitations demonstrated favorable short-term clinical and radiographic outcomes, in line with the growing body of evidence supporting the use of monolithic zirconia frameworks in full-arch implant dentistry [15,23,24,25].

### 4.1. Observed Associations

Independent of any mechanistic interpretation, the data-driven observations of this study can be summarized as follows. Mean MBR was higher in mandibular than maxillary arches (absolute difference 0.13 mm, d ≈ 2.77); within the mandible, V-shaped configurations showed higher MBR than U-shaped configurations (mean difference 0.10 mm, d ≈ 2.69); and inter-implant distance correlated strongly and negatively with MBR in the mandible (r = −0.70) but not in the maxilla (r = 0.01). All three associations showed large standardized effect sizes and were highly statistically significant (*p* < 0.001), although the corresponding absolute differences were numerically modest; the morphology and geometry associations remain exploratory, hypothesis-generating findings rather than confirmatory ones. Their consistency and statistical strength make a chance or borderline finding unlikely, but the present observational design cannot establish that arch anatomy independently modulates early MBR; these findings are best described as robust associations warranting mechanistic and confirmatory investigation in an independently powered, prospectively designed sample.

### 4.2. Biomechanical Hypothesis (Speculative Interpretation)

It is important to note that this study measured anatomical and geometric variables only (intercanine/intermolar width ratios, anterior arch angle, inter-implant distance, and cantilever length); no biomechanical parameter—such as mandibular flexure, functional strain, occlusal force, muscular activity, or stress distribution—was directly measured, and no finite element or other biomechanical analysis was performed. Accordingly, the associations reported here describe anatomy and geometry, not biomechanics, and the interpretation offered below is a hypothesis, not a finding.

The remainder of this discussion offers a biomechanical interpretation of the observed associations, which—while grounded in a robust and highly significant dataset—remains hypothetical. From this standpoint, the mandible differs from the maxilla in cortical thickness, trabecular organization, and capacity for elastic deformation under function [26]; when a rigid, continuous monolithic zirconia framework spans multiple implants, these characteristics could plausibly influence load transmission to peri-implant bone. The higher early MBR observed in V-shaped relative to U-shaped mandibles, together with the inverse correlation between inter-implant distance and MBR (r = −0.70)—that is, shorter inter-implant distances associated with higher MBR—would be compatible with, but does not demonstrate, a hypothesis in which narrower arch forms constrain implant distribution and are associated with increased local mechanical demand under a rigid framework [27]; this interpretation remains conjectural in the absence of direct biomechanical measurement. We emphasize that this remains an untested hypothesis: the present data cannot distinguish this mechanism from plausible alternative explanations, such as differences in bone quality, bone quantity, or soft-tissue thickness between morphological subgroups, and should not be read as evidence of mandibular flexure or stress distribution [28].

Beyond the anatomical and geometric hypothesis explored here, bone quality and implant positioning are independently established determinants of early MBR and merit explicit consideration as alternative or contributing explanations. Bone density and cortical thickness differ systematically between the maxilla and mandible, and higher bone quality has been associated with reduced marginal bone loss in prospective and cross-sectional studies [29]. Similarly, implant placement depth relative to the crestal bone level has been linked to differences in early marginal bone remodeling in some cohorts, although findings across studies are heterogeneous [30]. Because bone quality was not quantitatively assessed, insertion depth was not recorded as a discrete variable, and extraction status (immediate-extraction versus healed sites) was not analyzed as a potential confounder in the present study, we cannot exclude the possibility that some of the arch- and morphology-related differences reported here partly reflect these unmeasured factors rather than, or in addition to, the anatomical and geometric associations discussed above.

### 4.3. Clinical Implications

Although the arch- and morphology-related associations reported here are statistically robust and consistent across analyses, their clinical relevance should be interpreted cautiously. The observational design precludes causal inference, the underlying biomechanical mechanism was not directly measured (Section 4.2), and the absolute magnitude of the arch-level difference remains below the threshold conventionally considered clinically decisive after the first year of function (Section 3.1). Statistical robustness should therefore not be equated with clinical actionability: for most clinical scenarios, monolithic zirconia full-arch prostheses appear to provide predictable short-term outcomes in both arches, and the present findings do not, on their own, justify specific changes in implant planning, prosthetic design, or clinical practice.

These associations are best regarded as descriptive, hypothesis-generating observations that identify anatomical configuration as a variable worth monitoring and further investigating, rather than as a basis for prescriptive treatment recommendations. Any consideration of anatomical form during digital treatment planning should be viewed as exploratory until confirmed by adequately powered, pre-specified studies incorporating direct biomechanical assessment and controlling for the surgical, prosthetic, and bone-quality variables discussed in the Study Limitations.

### 4.4. Future Research

Longitudinal studies with extended follow-up periods beyond five years are needed to determine whether the early morphology- and geometry-related differences observed here persist, stabilize, or diminish over time.

Further research incorporating direct biomechanical measurements, such as strain gauge analysis or digital deformation tracking, would help clarify the mechanistic relationship between mandibular anatomy, prosthetic rigidity, and peri-implant bone response, and would help validate whether morphological classifications function as reliable clinical proxies for underlying biomechanical behavior.

Adequately powered, pre-specified, multicenter studies designed explicitly to confirm the magnitude and generalizability of the morphology and geometry associations identified here—ideally combined with three-dimensional morphological quantification and finite element modeling—would strengthen the evidence base before these findings are broadly translated into clinical decision-making.

Implant-level variability in early MBR—including whether posterior, tilted, or cantilever-adjacent implants exhibit disproportionate remodeling—is addressed in a companion publication [31] that analyzes the same underlying cohort of 40 patients and 49 prostheses (308 implants placed; 306 surviving at 12 months, reflecting two implant failures) using a three-level hierarchical (implant-within-arch-within-patient) model. The two studies pose distinct research questions using non-overlapping statistical approaches: the companion analysis partitions implant-level variance to identify which hierarchical level contributes most to overall MBR variability, without testing arch- or morphology-specific hypotheses, whereas the present study uses arch-level aggregation with a patient-clustering model to test whether MBR differs by arch and, exploratorily, by mandibular morphology and implant geometry. The two manuscripts should therefore be regarded as complementary analyses of the same dataset addressing different, non-overlapping questions rather than independent replications.

### 4.5. Study Limitations

Several limitations should be acknowledged. First, the observational design precludes causal inference regarding the relationship between arch morphology, implant geometry, and marginal bone remodeling. Second, mandibular flexure and strain were not directly measured, and morphology and geometric variables were used as clinically accessible proxies. Third, the 12-month follow-up reflects early remodeling and does not provide information on long-term bone stability.

Fourth, subgroup sample sizes for the morphology comparison were relatively small (*n* = 11–13 per group); although the observed effect was large and highly significant, replication in larger, independent samples remains desirable to confirm generalizability. Fifth, mechanical complications were rare and limited to two minor chipping events; consequently, no statistical inference regarding complication risk can be drawn from these observations.

Sixth, although the primary arch-level comparison accounted for within-patient clustering via a mixed-effects model, the morphology and geometry analyses were exploratory rather than confirmatory; while the very small *p*-values (*p* < 0.001) and large standardized effect sizes make a type I error unlikely to fully explain these findings, the absolute differences were numerically modest, and formal confirmation in adequately powered, prospectively designed, multicenter studies remains warranted to establish external validity.

Seventh, mandibular morphology was classified on the basis of alveolar ridge landmarks visible on CBCT rather than direct measurement of basal bone anatomy, and substantial but imperfect inter-examiner agreement (κ = 0.78) may have introduced non-differential misclassification, which would tend to attenuate rather than inflate the reported morphology-related differences. An additional limitation concerns the morphological classification itself: severe alveolar ridge resorption could alter intercanine/intermolar width ratios and the anterior arch angle independently of the patient’s original basal bone form, potentially reclassifying a basally U-shaped mandible as V-shaped (or vice versa) on the alveolar crest. This could introduce misclassification not captured by inter-examiner agreement alone, and future studies should consider basal bone contour (e.g., at the inferior border) as a complementary, resorption-resistant reference.

Eighth, primary implant stability was determined using insertion torque values alone; ISQ measurements via resonance frequency analysis were not performed, which limits comparability with studies using multimodal stability assessment and precludes any correlation analysis between RFA-based stability and early MBR.

Ninth, all surgeries were performed by a single experienced surgeon following a standardized protocol; while this minimizes inter-operator variability as a potential confounder, the influence of operator-related factors on early MBR could not be independently assessed.

Tenth, exact implant insertion depth (equicrestal vs. subcrestal) was not systematically recorded, precluding its analysis as a potential confounder of early MBR despite its established biological relevance.

Eleventh, bone quality, density, and cortical thickness were not quantitatively assessed at implant sites; these variables may represent important determinants of early MBR, and their absence limits our ability to disentangle anatomical (arch/morphology) effects from underlying bone-quality effects. Future studies should incorporate radiographic bone density assessment alongside morphological classification.

Twelfth, prosthetic design parameters—including occlusal vertical dimension, number of prosthetic units, implant and multi-unit abutment angulation, prosthetic screw-access position, and detailed opposing-dentition characteristics—were individualized per patient and were not captured as study variables; these factors may act as unmeasured confounders of the biomechanical interpretation offered in Section 4.2.

Thirteenth, both immediate-extraction and healed sites were included according to individual clinical indication but were not recorded as a discrete variable; no bone augmentation or guided bone regeneration procedures were performed in this cohort, as grafting was deliberately avoided to standardize surgical conditions, but extraction timing itself was not analyzed as a potential confounder.

Fourteenth, external validity is limited by the highly selective inclusion criteria of this cohort: a single implant system (bone-level), immediate loading only, monolithic zirconia frameworks only, a fully digital workflow, experienced clinicians, and a university-based clinical setting. These findings cannot necessarily be generalized to other implant systems, delayed-loading protocols, hybrid or segmented zirconia restorations, or routine private-practice settings, and should be confirmed in more heterogeneous, multicenter cohorts.

Despite these limitations, the use of standardized protocols, the prospective study design, the systematic geometric assessment, and the explicit accounting for within-patient clustering in the primary analysis contribute to the internal consistency and reliability of the reported observations.

## 5. Conclusions

Within the limitations of this prospective observational cohort study, and accounting for within-patient clustering in the primary analysis, monolithic zirconia full-arch prostheses demonstrated favorable short-term clinical and radiographic outcomes in both the maxilla and the mandible at 12 months. Statistically significant, highly consistent arch- and morphology-related associations with early marginal bone remodeling were observed between arches and, within the mandible, between U- and V-shaped configurations; these associations showed large standardized effect sizes, although the corresponding absolute differences were numerically modest and remained below the threshold conventionally considered clinically decisive after the first year of function. Given the observational design, these findings should be interpreted as associations rather than evidence of a causal or independently modulating effect of arch anatomy on bone remodeling, and the underlying biomechanical mechanism was not directly measured and remains hypothetical. Because no alternative framework material, stiffness, or strain was evaluated, the findings cannot be specifically attributed to the rigidity of monolithic zirconia and should not, on this basis, be extrapolated to other framework materials. Adequately powered, prospectively specified, multicenter studies incorporating direct biomechanical measurements and accounting for the surgical, anatomical, bone-quality, and prosthetic variables discussed in the Study Limitations are needed to confirm these associations, clarify their clinical significance, and elucidate their underlying mechanism.

## Figures and Tables

**Table 1:** Baseline Implant- and Prosthesis-Related Characteristics by Arch.

Variable	Maxilla (*n* = 25)	Mandible (*n* = 24)
Implants per arch, mean ± SD (range)	6.0 ± 0.8 (4–8)	6.6 ± 0.9 (4–8)
Anterior/posterior implant distribution, *n* (%)	88 (58.7%)/62 (41.3%)	92 (58.2%)/66 (41.8%)
Implant length, mean ± SD (mm)	11.3 ± 1.4	10.8 ± 1.2
Implant diameter, mean ± SD (mm)	4.1 ± 0.4	4.3 ± 0.4
Insertion torque, mean ± SD (range) (Ncm)	48.6 ± 8.1 (35–70)	52.4 ± 7.3 (35–75)
Arches fulfilling immediate loading criteria, *n* (%)	25 (100%)	24 (100%)
Opposing dentition (natural/prosthetic), *n* (%)	10 (40%)/15 (60%)	9 (37.5%)/15 (62.5%)
Relevant systemic/local risk factors, *n* (%)	6 (24%)	5 (20.8%)

**Table 2:** Implant and Prosthetic Survival Outcomes.

Outcome	Total (*n*)	Events (*n*)	Survival Rate ^1^ (%)
Implants placed	308	2 failures	99.4
Full-arch prostheses placed	49	1 failure	97.9

^1^ Calculated at the 12-month follow-up.

**Table 3:** Marginal Bone Remodeling (MBR) by Arch at 12 Months.

Arch	Number of Arches (*n*)	Mean MBR ± SD (mm)
Maxilla	25	0.73 ± 0.02 ^1^
Mandible	24	0.86 ± 0.06 ^1^

^1^ Mean MBR values represent the average of mesial and distal measurements per implant, averaged with equal weighting across implants within each arch (Section 2.7); implant counts per arch are reported in Table 1.

**Table 4:** Marginal Bone Remodeling (MBR) by Arch, Mandibular Morphology, and Implant Geometry.

Group	Total (*n*)	Mean MBR ± SD (mm)	Inter-Implant Distance ± SD (mm) ^2^	Cantilever Length ± SD (mm) ^3^
Maxilla	25	0.73 ± 0.02 ^1^	41.2 ± 1.0	11.4 ± 2.1
Mandible (overall)	24	0.86 ± 0.06 ^1^	37.5 ± 3.1	10.9 ± 2.3
Mandible (U-shaped)	13	0.81 ± 0.04 ^1^	39.7 ± 1.8	11.2 ± 2.0
Mandible (V-shaped)	11	0.91 ± 0.04 ^1^	35.0 ± 2.3	10.5 ± 2.5

^1^ Mean MBR values represent the average of mesial and distal measurements per implant, aggregated at the arch level. ^2^ Distance between the two most distal implants. ^3^ Distance from the distal implant to the prosthetic extension.

**Table 5:** Correlation between Geometric Variables and Marginal Bone Remodeling.

Arch	Variable	Pearson r	*p*-Value
Maxilla	Inter-implant distance	0.01	0.970
Maxilla	Cantilever length	0.09	0.631
Mandible	Inter-implant distance	−0.70	<0.001 *
Mandible	Cantilever length	0.18	0.394

* Exploratory, statistically significant correlation (*p* < 0.001); not corrected for multiplicity—see Section 2.7.

**Table 6:** Mechanical Complications by Arch and Mandibular Morphology.

Arch	Morphology	Type of Complication	Number of Events
Maxilla	-	None	0
Mandible	U-shaped	None	0
Mandible	V-shaped	Minor chipping *	2

* No framework fracture was observed.

**Table 7:** Patient-Reported Outcomes at Baseline and 12 Months.

Outcome Measure	Baseline Mean ± SD	12 Months Mean ± SD	*p*-Value
OHIP-14 score ^1^	52.3 ± 8.1	18.9 ± 4.3	<0.001
VAS satisfaction ^2^	4.1 ± 1.2	9.2 ± 0.7	<0.001

^1^ Lower OHIP-14 scores indicate improved oral health-related quality of life. ^2^ Higher VAS scores indicate greater patient satisfaction.

## Data Availability

The data presented in this study are not publicly available due to privacy and ethical restrictions related to clinical patient data. De-identified data may be made available by the corresponding author upon reasonable request, subject to institutional approval and applicable data protection regulations. The R code (version 4.4.1; R Core Team, Vienna, Austria) used for the statistical analysis is also available from the corresponding author upon reasonable request.

## Supplementary Materials

The following supporting information can be downloaded at: https://www.mdpi.com/article/10.3390/bioengineering13080933/s1, Supplementary File S1: The STROBE (Strengthening the Reporting of Observational Studies in Epidemiology) checklist for cohort studies, completed for this manuscript.

## Abbreviations

The following abbreviations are used in this manuscript:

MBR	Marginal Bone Remodeling
CBCT	Cone-Beam Computed Tomography
CAD	Computer-Aided Design
CAD/CAM	Computer-Aided Design/Computer-Aided Manufacturing
STROBE	Strengthening the Reporting of Observational Studies in Epidemiology
ICC	Intraclass Correlation Coefficient
PMMA	Polymethyl Methacrylate
3Y-TZP	3 mol% Yttria-Stabilized Tetragonal Zirconia Polycrystal
ANOVA	Analysis of Variance
SD	Standard Deviation
CI	Confidence Interval
CMOS	Complementary Metal-Oxide Semiconductor
OHIP-14	Oral Health Impact Profile-14
VAS	Visual Analogue Scale
