# Supplementary material for: Arch- and Morphology-Related Variation in Early Marginal Bone Remodeling of Monolithic Zirconia Full-Arch Prostheses: An Exploratory Prospective Cohort Study
Source: Bioengineering (Basel). 2026 Aug 18;13(8):933. doi: 10.3390/bioengineering13080933 (PMC13510003; doi:10.3390/bioengineering13080933)
Supplement: Supplementary file 1 [file bioengineering-13-00933-s001.zip › File S1/Carta aprovació REST-ECL-2020-02 Luis Carlos Garza.pdf]

APROVACIÓ ESTUDI PEL CEIC / APROBACIÓN ESTUDIO POR EL CEIC / RESEARCH ETHICAL COMMITTEE APPROVAL STUDY

Codi de l'estudi / Código del estudio / Study Code:
Versió del protocol/ Versión del protocolo / Study version: 1.2
Data de la versió/ Fecha de la versión/ Version date: 17/09/20
Títol/ Título / Title: Study of hard and soft tissue behaviour around abutments on implants
Investigador Principal / Main researcher: Luis Carlos Garza
Tutor/Monitor: Dr. Miguel Roig
Investigador/a Secundario/a: Ricardo David Palacios

Sant Cugat del Vallès, 29 de setembre de 2020

Benvolgut Doctor,

Els membres del CEIm de la Clínica Universitària d'Odontologia de la UIC, els hi agraeixen l'aportació científica en el camp de la investigació i la presentació del Protocol en aquest Comitè per a la seva avaluació.

Valorades les noves aportacions realitzades a l'estudi, sol·licitades pel nostre CEIm, el dia 19 de setembre de 2020, li comuniquem que el dictamen final ha sigut FAVORABLE.

Li informem que s'haurà de presentar al Comitè d'Ètica d'investigacions amb medicaments, i a través de la Comissió Científica, un informe preliminar anual del seguiment de l'estudi i un informe final un cop finalitzat aquest.

El Comitè, tant en la seva composició, com en els PNT, compleix amb les normes de BPC (CPMP/ICH/135/95) i amb el Real Decret 1090/2015, i la seva composició actual és la següent:

- Dr. J.Manuel Ribera Uribe (Presidente, Medico-estomatólogo)
- Dr. Pau Ferrer Salvans (Vicepresidente, Farmacólogo clínico)
- Sra. Noelia Nogales (Secretaria técnica, Bióloga)
- Dr. Joan Janáriz Roldán (Miembro, Médico especialista en medicina interna i oncología)
- Dr. Andreu Hernando Chaure (Miembro, Jurista)
- Sra. Patricia Dominguez Tordera (Miembro, Farmacéutica Hospitalaria)
- Sra. Klaudia Obolоñczyk (Miembro, Farmacéutica de Atención Primaria)
- Dr. Christian Villavicencio-Chávez (Miembro, Médico gerontólogo)
- Sra. Laia Wennberg Capellades (Miembro, Enfermera)
- Sr. Antonio Alcáraz Gibert (Miembro lego, Persona ajena a la profesión sanitaria)
- M. Carmen García Monge (Miembro, Unidad de atención al usuario)

Que en aquesta reunió del Comitè Ètic d'Investigació Clínica amb medicaments es va cumplir amb el quorum preceptiu legalment.

Atentament,

Apreciados Doctores,

Los miembros del CEIm de la Clínica Universitària d'Odontologia de la UIC, les agradecen su aportación científica en el campo de la investigación y la presentación del Protocolo a este Comité para su evaluación.

Valoradas las nuevas aportaciones realizadas al estudio, solicitadas por nuestro CEIm, el 19 de septiembre de 2020, le comunicamos que el dictamen final ha sido FAVORABLE.

Le recordamos que deberá presentar al Comitè d'Ètica d'Investigacions amb medicaments de la CUO, y a través de la Comisión Científica, un informe preliminar mensual del seguimiento del estudio y un informe final una vez finalizado el mismo.

Atentamente,

Dear Doctors,

The members of the CEIm of the Clínica Universitària d'Odontologia, appreciate your contribution in the field of research and the presentation to this Committee of the referred study for its evaluation.

After having rated the new contributions to the study, requested by our Ethic Committee, on 19 September 2020, the decision was to APPROVE it.

We remind that you should present a monthly preliminary report during the study and a final report when the study finishes, through the Academic Commission, to the Drug Research Ethics Committee.

Best regards,

RIBERA URIBE JOSE MANUEL - 37314164E

Firmado digitalmente por RIBERA URIBE JOSE MANUEL - 37314164E
Fecha: 2020.09.30 10:02:15 +02'00'

Dr. J.Manuel Ribera
President CEIm
