# Supplementary material for: Arch- and Morphology-Related Variation in Early Marginal Bone Remodeling of Monolithic Zirconia Full-Arch Prostheses: An Exploratory Prospective Cohort Study
Source: Bioengineering (Basel). 2026 Aug 18;13(8):933. doi: 10.3390/bioengineering13080933 (PMC13510003; doi:10.3390/bioengineering13080933)
Supplement: Supplementary file 1 [file bioengineering-13-00933-s001.zip › File S1/Ethics committee approval letter.pdf]

Universitat Internacional de Catalunya

Comitè d'Ètica d'Investigació amb medicaments

uic barcelona

RESEARCH ETHICAL COMMITTEE APPROVAL STUDY

Study Code: REST-ECL-2020-02
Study version: 1.2
Version date: 17/09/20
Title: Study of hard and soft tissue behaviour around abutments on implants
Main researcher: Luis Carlos Garza
Monitor: Dr. Miguel Roig
Secondary researcher: Ricardo David Palacios

Sant Cugat del Vallès; September 29, 2020

Dear Doctor,

The members of the CEIm (Clinical Research Ethics Committee) at the University Dental Clinic of UIC would like to thank you for your scientific contribution to the field of research and for submitting the protocol to this Committee for evaluation.

Having reviewed the new contributions made to the study, as requested by our CEIm on 19 September 2020, we inform you that the final opinion is **FAVOURABLE**.

We would like to inform you that a preliminary annual follow-up report of the study, as well as a final report upon its completion, must be submitted to the Ethics Committee for Research with Medicines through the Scientific Committee.

The Committee, both in its composition and in its Standard Operating Procedures (SOPs), complies with Good Clinical Practice (CPMP/ICH/135/95) guidelines and with Royal Decree 1090/2015. Its current composition is as follows:

- Dr. J. Manuel Ribera Uribe (President, Physician-Stomatologist)

- Dr. Pau Ferrer Salvans (Vice President, Clinical Pharmacologist)

- Ms. Noelia Nogales (Technical Secretary, Biologist)

- Dr. Joan Janáriz Roldán (Member, Specialist in Internal Medicine and Oncology)

- Dr. Andreu Hernando Chaure (Member, Legal Expert)

• Ms. Patricia Dominguez Tordera (Member, Hospital Pharmacist)

• Ms. Klaudia Oboloñczyk (Member, Primary Care Pharmacist)• Dr. Christian Villavicencio-Chávez (Member, Geriatric Physician)

• Ms. Laia Wennberg Capellades (Member, Nurse)

• Mr. Antonio Alcáraz Gibert (Lay Member, Person outside the healthcare

profession)

• Ms. M. Carmen García Monge (Member, User Support Unit)

This meeting of the Clinical Research Ethics Committee with Medicines met the legally required quorum.

Best regards,

RIBERA URIBE JOSE MANUEL - 37314164E

Firmado digitalmente por RIBERA URIBE JOSE MANUEL - 37314164E
Fecha: 2020.09.30 10:02:15 +02'00'

Dr. J.Manuel Ribera
President CEIm
