# Supplementary material for: Arch- and Morphology-Related Variation in Early Marginal Bone Remodeling of Monolithic Zirconia Full-Arch Prostheses: An Exploratory Prospective Cohort Study
Source: Bioengineering (Basel). 2026 Aug 18;13(8):933. doi: 10.3390/bioengineering13080933 (PMC13510003; doi:10.3390/bioengineering13080933)
Supplement: Supplementary file 1 [file bioengineering-13-00933-s001.zip › File S1/STROBE_checklist_completed.pdf]

STROBE Statement — Checklist of items that should be included in reports of cohort studies

Manuscript: Arch- and Morphology-Related Variation in Early Marginal Bone Remodeling of Monolithic Zirconia Full-Arch Prostheses: An Exploratory Prospective Cohort Study

Topic	Item No	Recommendation	Reported in manuscript (section)
Title and abstract	1a	Indicate the study's design with a commonly used term in the title or the abstract	Title ("...Prospective Cohort Study"); Abstract
	1b	Provide in the abstract an informative and balanced summary of what was done and what was found	Abstract (full)
Introduction			
Background/rationale	2	Explain the scientific background and rationale for the investigation being reported	Introduction, §1–7
Objectives	3	State specific objectives, including any prespecified hypotheses	Introduction, final paragraph ("Therefore, the objectives...")
Methods			
Study design	4	Present key elements of study design early in the paper	Section 2.1
Setting	5	Describe the setting, locations, and relevant dates, including periods of recruitment, exposure, follow-up, and data collection	Section 2.2
Participants	6a	Give the eligibility criteria, and the sources and methods of selection of participants. Describe methods of follow-up	Section 2.2 (eligibility); Section 2.5 (follow-up assessment)
	6b	For matched studies, give matching criteria and number of exposed and unexposed	Not applicable – not a matched study
Variables	7	Clearly define all outcomes, exposures, predictors, potential confounders, and effect modifiers. Give diagnostic criteria, if applicable	Sections 2.2, 2.5, 2.6
Data sources/measurement	8*	For each variable of interest, give sources of data and details of methods of assessment (measurement). Describe comparability of assessment methods if there is more than one group	Sections 2.5, 2.6, 2.2
Bias	9	Describe any efforts to address potential sources of bias	Sections 2.5, 2.6; Discussion §4.5
Study size	10	Explain how the study size was arrived at	Section 2.7 (sample size calculation)
Quantitative variables	11	Explain how quantitative variables were handled in the analyses. If applicable, describe which groupings were chosen and why	Section 2.6 (U-/V-shape cut-offs); Section 2.7
Statistical methods	12a	Describe all statistical methods, including those used to control for confounding	Section 2.7
	12b	Describe any methods used to examine subgroups and interactions	Section 2.7
	12c	Explain how missing data were addressed	Results, (no missing data: completeness 100%)
	12d	If applicable, explain how loss to follow-up was addressed	NA
	12e	Describe any sensitivity analyses	Section 2.7; Results §3.1 (dual-arch sensitivity analysis)
Results			
Participants	13a	Report numbers of individuals at each stage of study—eg numbers potentially eligible, examined for eligibility, confirmed eligible, included in the study, completing follow-up, and analysed	NA
	13b	Give reasons for non-participation at each stage	Not reported
	13c	Consider use of a flow diagram	Not included
Descriptive data	14a	Give characteristics of study participants (eg demographic, clinical, social) and information on exposures and potential confounders	Table 1
	14b	Indicate number of participants with missing data for each variable of interest	Results, §3 (data completeness 100%)
	14c	Summarise follow-up time (eg, average and total amount)	Results, §3 (fixed 12-month follow-up for all participants)
Outcome data	15*	Report numbers of outcome events or summary measures over time	Results §3, 3.1, 3.2, 3.3; Tables 2, 3, 6, 7

Topic	Item No	Recommendation	Reported in manuscript (section)
Main results	16a	Give unadjusted estimates and, if applicable, confounder-adjusted estimates and their precision (eg, 95% CI). Make clear which confounders were adjusted for and why	Section 3.1; Tables 3–5
	16b	Report category boundaries when continuous variables were categorized	Section 2.6
	16c	If relevant, consider translating estimates of relative risk into absolute risk for a meaningful time period	Not applicable – no relative-risk outcome in this study
Other analyses	17	Report other analyses done—eg analyses of subgroups and interactions, and sensitivity analyses	Section 3.1
Discussion			
Key results	18	Summarise key results with reference to study objectives	Discussion, opening paragraph; Section 4.1
Limitations	19	Discuss limitations of the study, taking into account sources of potential bias or imprecision. Discuss both direction and magnitude of any potential bias	Section 4.5
Interpretation	20	Give a cautious overall interpretation of results considering objectives, limitations, multiplicity of analyses, results from similar studies, and other relevant evidence	Sections 4.1, 4.2, 4.3
Generalisability	21	Discuss the generalisability (external validity) of the study results	Not explicitly addressed as a standalone point
Other information			
Funding	22	Give the source of funding and the role of the funders for the present study and, if applicable, for the original study on which the present article is based	"Funding" statement (end of manuscript)

** Give information separately for exposed and unexposed groups (in this manuscript: maxillary vs. mandibular arches, reported in Table 1).*

Note: An Explanation and Elaboration article discusses each checklist item and gives methodological background and published examples of transparent reporting. The STROBE checklist is best used in conjunction with this article (freely available on the Web sites of PLoS Medicine, Annals of Internal Medicine, and Epidemiology). Information on the STROBE Initiative is available at www.strobe-statement.org.
